# User Friendliness and Perioperative Guidance Benefits of a Cataract Surgery Education App: Randomized Controlled Trial

**DOI:** 10.2196/55742

**Published:** 2024-03-29

**Authors:** Rokas Gerbutavicius, David A Merle, Armin Wolf, Spyridon Dimopoulos, Karsten Ulrich Kortuem, Friederike Charlotte Kortuem

**Affiliations:** 1 Department for Ophthalmology University of Tuebingen Tuebingen Germany; 2 University Eye Hospital University of Ulm Ulm Germany; 3 Institute for Ophthalmic Research Center for Ophthalmology University of Tuebingen Tuebingen Germany; 4 Department of Ophthalmology Medical University of Graz Graz Austria; 5 Augenarztpraxis Dres. Kortuem Ludwigsburg Germany

**Keywords:** mHealth, mobile health, workflow optimization, patient satisfaction, health education, educational background, phacoemulsification

## Abstract

**Background:**

Cataract surgeries are among the most performed surgeries worldwide. A thorough patient education is essential to inform patients about the perioperative process and postoperative target results concerning the intraocular lens and objectives for visual outcomes. However, addressing all relevant aspects and questions is time-consuming. Mobile apps can facilitate this process for both patients and physicians and thus be beneficial. However, the success of such an app depends on its user friendliness and acceptance by patients.

**Objective:**

This study aimed to evaluate the user friendliness and acceptance of a cataract surgery education app on mobile devices among patients undergoing cataract surgery, the characteristics of patients who benefit the most from app use, and the influence of the app on patient satisfaction with treatment.

**Methods:**

All patients who underwent cataract surgery at an ophthalmological practice from August 2020 to July 2021 were invited to participate in this randomized controlled trial. Out of 493 invited patients, 297 (60.2%) were enrolled in this study. Patients were randomized into 3 different groups. Half of the patients were offered to participate in Group 1 with use of the “Patient Journey” app. However, if they decided not to use the app, they were included in Group 2 (app denial). The other half of the patients were included in Group 3 (control) with no use of the app and with information provided conventionally. The app provided general information on the ophthalmological center, surgeons, cataract, and treatment options. Different questionnaires were used in all 3 groups to evaluate satisfaction with the perioperative process. Group 1 evaluated the app. Demographic characteristics, such as age, gender, and educational degree, were assessed.

**Results:**

Group 1 included 77 patients (median age 69 years). Group 2 included 61 patients, and their median age was higher (median age 79 years). Group 3 included 159 patients (median age 74 years). There was no difference in satisfaction with the perioperative process and clinic between the 3 groups. Almost all app users appreciated the digital details provided for the organization and the information on the surgery. Age did not play a major role in appreciation of the app. Female patients tended to appreciate the information provided more than male patients. Patients who did not have a higher university degree experienced more benefits from the informational content of the app and were the most satisfied with the information. However, male patients and academics were in general more aware of technology and handled the app more easily.

**Conclusions:**

The app showed high user friendliness and acceptance, and could particularly benefit specific patient groups. App users demonstrated a noninferior high satisfaction with the treatment in the ophthalmological center in comparison with patients who were informed about the surgery only conventionally.

## Introduction

### Changes in Health Care Toward Digitalization

Health care is currently undergoing a substantial transition due to technological progress that reshapes clinical workflows. The progress in digitalization opens the door for new ways to organize, diagnose, educate, and treat patients. In 2023, the number of smartphone end users reached almost 7 billion globally, which equals roughly 86% of the world’s population [1]. Compared to 7 years earlier, these numbers almost doubled (3.7 billion or 49.4% in 2016) [1]. Intriguingly, mobile phone–based apps can provide valuable new platforms to address patients’ and physicians’ needs. In recent years, many apps in health care were developed driven by an increasing public interest in digital health care products. In 2017 alone, 3.7 billion mobile health (mHealth) app downloads were counted worldwide [2], showing a continuous almost exponential growth over the last few years. Health apps on mobile devices, also known as mHealth apps, are global software programs that can be used by patients, health care professionals, or other care givers. In Germany, since the end of 2019, the Digital Health Care Act (Digitales Versorgungsgesetz, DVG) allows app developers to enter a process at a governmental agency to receive reimbursement for the download and use of the app from statutory health insurances. This has given another boost to the development of apps with greater and proven quality [3].

### mHealth in Ophthalmology

In March 2020, a review identified 131 ophthalmology-related mobile apps, with 32% of the apps designed for visual acuity testing and screening, 13% designed for eye relaxation exercises, and 12% designed for professional training. The remaining apps included tools to detect color blindness, tools that served as low vision aids, or tools aimed to provide assistance and patient education. Strikingly, less than 5% were documented to have been tested for validity [4].

A recent review by Nagino et al [5] summarized published data on the clinical utility of 48 mobile apps in ophthalmology. Of those, 35% supported clinical ophthalmological examination, 27% intended to detect ophthalmological diseases, 20% supported medical personnel, 10% informed patients about ophthalmological diseases, and 6% were designed to encourage compliance. Only 2 apps reported significant efficacy in treating diseases [5]. An app for glaucoma treatment reminded patients to administer intraocular pressure (IOP) lowering eye drops [6]. Patients enrolled in the study showed higher adherence when being reminded by the app. The IOP lowering effect was however not significant in any of the subgroups [6]. Patients with macular diseases that required regular injections benefited in terms of visual acuity when provided with mobile hyperacuity home monitoring via an app and discontinued treatment less often [7].

However, most health apps in ophthalmology and other disciplines still lack evidence of clinical effectiveness and thus lack the rational basis to qualify for reimbursement [8,9]. For the vast majority of apps available in digital app stores, no published scientific data or proof of efficacy is available.

On the other hand, digital apps hold great potential value for health care providers in optimizing work processes and flows. Additionally, in light of the looming physician shortage and ever-increasing patient consultations, apps can be valuable tools to offer new ways of interaction between patients and physicians and can add value by improving patient education [10].

### Cataract Surgery

Cataract is a very common condition that is highly associated with aging and ultimately occurs in every individual. The initial symptoms include reduced visual acuity and increased glare sensitivity. Depending on the localization of increased opacity, cataracts can be divided into nuclear, cortical, or subcapsular cataracts. In an observational study by Klein et al [11], the cumulative incidence of nuclear cataract increased from 2.9% in persons aged 43 to 54 years at baseline to 40% in those aged 75 years or older. For cortical and posterior subcapsular cataract, the corresponding values were 1.9% and 21.8% and 1.4% and 7.3%, respectively. These numbers illustrate the disease burden and high number of patients experiencing the consequences of cataract. Accordingly, cataract surgery is ranked as the most common surgical procedure performed in the European union, with almost 5 million surgeries performed in 2017 alone. Multiple studies have demonstrated gains in visual function and quality of life after surgery [12-14]. The standard technique in Europe is the removal of the opacified lens by phacoemulsification and the implantation of an artificial intraocular lens (IOL). The surgery is mostly conducted under topical or peribulbar anesthesia [15,16]. In complicated cases, general anesthesia or the use of short-acting sedatives may be necessary. Although phacoemulsification was the standardized procedure for a long time, novel approaches that allow better precision in incisions and fragmentation of the lens have emerged. In this light, laser-supported phacoemulsification, such as nanosecond and femtosecond laser, promise better accuracy and less strain on the cornea without any effect on visual outcomes [17-19]. Furthermore, new developments in IOL design have led to a vast selection of different available lenses, such as multifocal or enhanced depth of focus lenses, possibly increasing quality of life [20]. With the obvious plethora of different options, patients frequently feel overwhelmed regarding the choice of the perioperative process and the targeted refraction (monofocal near or distance vision, and multifocal satisfactory near, intermediate, or distance vision). These questions and decisions possibly involve higher out-of-pocket costs for the patient as newer techniques and IOL designs are often not covered by statutory or private health insurances. Furthermore, multifocal lenses are also known to have potential negative aspects due to phenomena such as glare, halos, and loss of contrast sensitivity. This may lead to unsatisfactory results in some patients and thus require highly accurate refractive outcomes, including limited astigmatism, in order to achieve good function and postoperative tolerance [21]. To ensure good outcomes and patient satisfaction, the treating ophthalmologist must provide sufficient information and time to the patient, provide guidance throughout the process, and carefully select patients suitable for implantation of specific lenses.

### Perioperative Journey of Patients

As for every intervention or surgery, the patient’s journey is linked to uncertainty and a certain degree of anxiety. For fields other than ophthalmology, mHealth apps seem to be acknowledged by patients in a perioperative setting. In studies with mobile apps, patients felt more taken care of, with a clear focus on the patient’s satisfaction and health. Furthermore, the apps proved to be more cost-effective and efficient in health care services [22,23]. Likewise, a recent study was able to improve the adherence of cataract patients to postoperative management through the use of reminder messages on a mobile app, which also provided links to educational videos online [24].

However, there is no published study on patients undergoing cataract surgery who have been guided by a mobile app throughout the perioperative process as a whole. Therefore, this study aimed to assess the acceptance and satisfaction with a mobile app accompanying the patients in their cataract surgery journey. As cataract patients are mostly in the second half of their life, this investigation addresses the use of mHealth in an elderly population. Additionally, the study also addresses the question of which group of patients benefits the most from app use.

## Methods

### Study Patients

All patients who presented to a local ophthalmological practice center and underwent cataract surgery over a period of 1 year (August 2020 to July 2021) were offered to participate in this prospective, single-center, randomized controlled trial. Invitations to the trial were sent by mail to the patients 2 to 3 weeks prior to the pre-examination day. Invitations comprised relevant information on the trial and a consent sheet for participation and data. Out of 493 patients who received an invitation to participate, 297 (60.2%) were willing to be enrolled in this study. Over the duration of the study, of the 297 patients, 181 (60.9%) were operated on 1 eye and 116 (39.1%) were operated on both eyes. Patients who consented were randomized by even and uneven patient numbers issued by the health care information system (software) into 3 groups as follows: (1) Patients with even patient numbers were offered to participate in the interventional group (Group 1) with the use of the mobile app; (2) Patients who did not want to use the app were included in the app deny group (Group 2); (3) Patients with uneven patient numbers were included in the control group (Group 3) without access to the app. Group 1 received personal login details and information on the download and use of the app.

### App Description

The app used in this trial was the “Patient Journey” app (Versions 4.15.0, 4.26.8, and 4.30.0; Interactive Studios). The app can be installed on all devices with iOS (Apple) and Android (Google) operating systems. The app was free for all participants.

**Figure 1:**
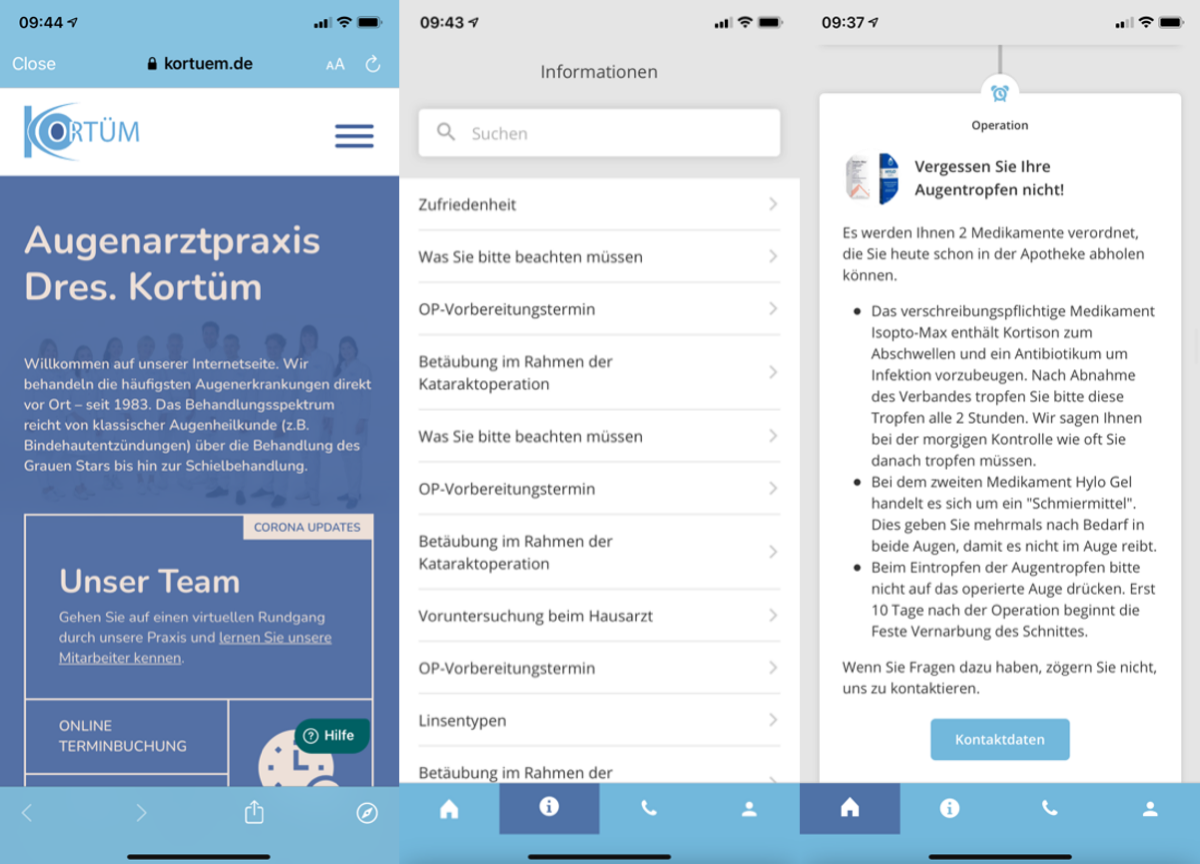
Screenshots of the Patient Journey app with center information, patient information, and postoperative recommendations on the use of ocular drops.

The app uses an adjustable content management system with the possibility of own branding. The app versions used in this study had the following 4 sections: (1) General information on the ophthalmological center and surgeons along with contact information; (2) Information on appointments (surgery, and preoperative and postoperative follow-ups); (3) Information about the disease (cataract), treatment, anesthesia options, and IOL; and (4) Preoperative, perioperative, and postoperative behavioral recommendations and the medication treatment scheme (Figure 1).

In the third section, a video on cataract development, symptoms, and therapy was available. This video was almost 3 minutes long and included real ophthalmologists and patients who were interviewed in a hospital. Furthermore, pictures and text in the app provided information on IOLs, anesthetic procedures, and postoperative care. Questionnaires were included in order to find a suitable IOL and target refraction. Early preoperative instructions comprised information about necessary blood tests, the need to schedule an appointment with the anesthesiologist in the case of surgery under general anesthesia, and the prohibition to directly drive a car after the pre-examination visit owing to mydriasis. One day before the surgery, patients received information about the time, location, and course of the surgery, and in the case of general anesthesia, they received information about the necessity of preoperative fasting. On the surgery date, patients were, if needed, reminded to continue preoperative fasting and instructed about postoperative behaviors, including intake of medications, wearing of an eye patch, taking a shower, performing physical activity, and observing possible symptoms like pain or foreign body sensation. Postoperative care in the app comprised information about scheduled appointments in the practice as well as about common and warning postoperative symptoms, including their management or the necessity to schedule an additional examination. Push notifications could be set to remind the patient to apply anti-inflammatory drops or to keep the appointments.

Study organizers stayed in contact with patients from all 3 study groups on a regular basis from invitation to the study 2 to 3 weeks before the pre-examination visit until 4 to 6 weeks after the first cataract surgery and after the second cataract surgery, if performed. Patients in all study groups communicated with the practice and were informed about the surgery conventionally while attending ophthalmologist appointments or via a phone call. App users additionally received necessary information about the surgery and could schedule appointments or contact the practice over the app. Patients who could not manage to install the app on their own were offered support for the installation and set-up of the app in the practice at the pre-examination visit or via a phone call. See Multimedia Appendix 1 for a demonstration video of the cataract app.

### Patient Survey

For the study, 6 different nonvalidated digital questionnaires were developed by study organizers to address the characteristics of the participating patients and their satisfaction with the app and the practice. Several validated questionnaires were taken into account for the development of these questionnaires [10,25-28]. Groups 1 and 2 had to fill out 3 questionnaires, while Group 3 had to fill out 2 questionnaires. Please change to: "All 3 groups were asked to provide answers to questions regarding satisfaction with the practice. Group 1 additionally had questionnaires that addressed the app (installation and handling), previous use of other health care apps, and the highest educational degree. Group 2 was also asked about the reasons for denying to use the app, as well as about previous use of other health care apps and the highest educational degree. Group 3 provided answers to questions regarding previous experience with other health care apps and the highest educational degree. All questionnaires were electronically accessible and could be completed on a webpage of the app developer Interactive Studios (Table 1). See Multimedia Appendix 2 for the questionnaires used in the study groups.

**Table 1:** Study questionnaires in the course of treatment.

Timeline of the questionnaires	Group 1 (app use)	Group 2 (app denial)	Group 3 (control)
Surgery pre-examination appointment	Questionnaire E (satisfaction with app installation and general information about the patient)	Questionnaire B (reasons for denying app use) Questionnaire F (general information about the patient)	Questionnaire F (general information about the patient)
1-7 days after surgery	Questionnaire A (satisfaction with the app)	No questionnaire	No questionnaire
4-6 weeks after surgery	Questionnaire C (satisfaction with the app and practice)	Questionnaire D (satisfaction with the practice)	Questionnaire D (satisfaction with the practice)

Patients who could not fill out the questionnaires online on their own were offered personal support in the practice or via a phone call. Demographic characteristics, including age, gender, and prior cataract surgery, were extracted from medical health records available in the practice. App use data of the entire cohort (Group 1) were collected by the app developer and could be accessed online by the study organizers. Individual personal data were not attainable due to data privacy.

The collected data were subjected to descriptive and quantitative analyses using IBM SPSS Statistics 27 (IBM Corp). Statistical significance was reached at α=.05.

### Ethical Considerations

This study was approved by the Ethics Committee of Landesaerztekammer Baden-Wuerttemberg (Stuttgart, Germany; approval number: F-2021-004) as a study involving human subjects and followed the ethical considerations of the Declaration of Helsinki. All participants were thoroughly informed about the study, and if approved, they signed an informed consent form allowing research on the gained data. All obtained data were deidentified, and no images allowing reidentification of the subjects were included. Participants did not receive any monetary compensation. No generative artificial intelligence system was used in any portion for manuscript writing.

## Results

### Demographics of the Groups

The study included 297 patients who underwent cataract surgery. Of the 297 patients, 77 (25.9%) used the app (Group 1), 61 (20.6%) did not want to use the app (Group 2), and 159 (53.5%) were included in the control group (Group 3). In all groups, more female patients were included (Table 2). In Groups 1, 2, and 3, the median patient ages were 69, 79, and 74 years, respectively. The mean patient age was significantly higher in Group 2 than in the other 2 groups (Kruskal-Wallis test, *P*<.001). All patients were asked for their highest degree of education. In Group 1, most patients had completed a professional apprenticeship or a higher university degree. In Group 2, only 8 patients had a higher educational degree. This difference between groups approached significance (chi-square test, *P*=.05). The majority of patients had surgery on only 1 eye.

**Table 2:** Demographics of the patients in the study groups.

Study participants		Group 1 (app use) (n=77)	Group 2 (app denial) (n=61)	Group 3 (control) (n=159)	*P* value^a^
**Sex, n (%)**					.77^b^
	Male	34 (44.2)	24 (39.3)	71 (44.7)	
	Female	43 (55.8)	37 (60.7)	88 (55.3)	
**Age**					<.001^c^
	Median (minimum-maximum)	69 (50-86)	79 (61-88)	74 (47-94)	
	Mean	69.2	77.6	72.7	
Experience with health apps, n (%)		20 (25.9)	3 (5.8)	23 (16.3)	.01^b^
**Highest degree of education, n (%)**					.06^b^
	School degree	23 (29.9)	21 (40.4)	63 (45.0)	
	Professional apprenticeship	29 (37.7)	23 (44.2)	51 (36.4)	
	Higher university degree	25 (32.5)	8 (15.4)	26 (18.6)	
**Eyes undergoing surgery, n (%)**					.86^b^
	One eye	46 (59.7)	39 (63.9)	96 (60.4)	
	Both eyes	31 (40.3)	22 (36.1)	63 (39.6)	

^a^*P* value is the probability of rejecting the correct null hypothesis.

^b^*P* value was calculated using the chi-square test.

^c^*P* value was calculated using the nonparametric Kruskal-Wallis test.

### Characteristics of Group 2 (App Denial)

Patients who dismissed the app were asked for their reasons. Most (41/61, 67%) answered that they were missing a suitable device. Moreover, 9 patients reported no interest or a lack of technological competence for the use of the app. Two patients mentioned insufficient vision. Most (49/52, 94%) of the patients in this group had no previous experience with health care apps. Group 3 had a relatively high percentage of patients (23/141, 16.3%) who had previous experience. Moreover, in Group 1, 26% (20/77) of patients already had an experience with health apps (chi-square test, *P*=.01).

### Patient Satisfaction With the Ophthalmological Center During Cataract Surgery

After 4 to 6 weeks, patient satisfaction with the ophthalmological center where they had their surgery was evaluated in all groups (Table 3). In Group 1, 65% (46/71) were very satisfied, 34% (24/71) were fairly satisfied, and 1% (1/71) were unsatisfied. Similar responses were provided in the other 2 groups. In Group 2, 67% (40/60) were very satisfied and 32% (19/60) were fairly satisfied. In Group 3, 66.9% (105/157) were very satisfied and 31.2% (49/157) were fairly satisfied.

**Table 3:** Comparison of groups regarding satisfaction with the center and possible areas for improvement within the ophthalmological center.

Patient satisfaction		Group 1 (app use), n/N (%)	Group 2 (app denial), n/N (%)	Group 3 (control), n/N (%)	*P* value^a^
**Patient overall satisfaction with the center**					.99^a^
	Very satisfied	46/71 (64.8)	40/60 (66.7)	105/157 (66.9)	
	Fairly satisfied	24/71 (33.8)	19/60 (31.7)	49/157 (31.2)	
	Unsatisfied	1/71 (1.4)	1/60 (1.7)	3/157 (1.9)	
**I was *extensively* counseled and informed**					.97^a^
	Agree completely	49/73 (67.1)	37/59 (62.7)	103/157 (65.6)	
	Agree	21/73 (28.8)	20/59 (33.9)	47/157 (29.9)	
	Do not agree (partly)	3/73 (4.1)	2/59 (3.4)	7/157 (4.5)	
**I was *comprehensibly* counseled and informed**					.89^a^
	Agree completely	51/73 (69.9)	40/59 (67.8)	108/158 (68.4)	
	Agree	19/73 (26.0)	17/59 (28.8)	40/158 (25.3)	
	Do not agree (partly)	3/73 (4.1)	2/59 (3.4)	10/158 (6.3)	
**I was friendly and attentively treated**					.20^a^
	Agree completely	48/73 (65.8)	43/59 (72.9)	118/158 (74.7)	
	Agree	19/73 (26.0)	14/59 (23.7)	37/158 (23.4)	
	Do not agree (partly)	6/73 (8.2)	2/59 (3.4)	3/158 (1.9)	
**Areas for improvement (multiple answers possible)**					.22^a^
	Center’s organization	20/76 (26.3)	23/65 (35.4)	51/165 (31.1)	
	Infrastructure	11/76 (14.5)	8/65 (12.3)	26/165 (25.9)	
	Communication	9/76 (11.8)	3/65 (4.6)	5/165 (3.0)	
	Counseling and information	5/76 (6.6)	7/65 (10.8)	10/165 (6.1)	
	No suggestions	31/76 (40.8)	24/65 (36.9)	72/165 (43.9)	

^a^*P* value was calculated using the chi-square test.

Most patients felt counseled and informed comprehensibly (Group 1: 70/73, 95.9%; Group 2: 57/59, 96.6%; Group 3: 148/158, 93.7%) and in detail (Group 1: 70/73, 95.9%; Group 2: 57/59, 96.6%; Group 3: 150/157, 95.5%). Over 90% of patients in all groups completely agreed or agreed in this matter (Table 3). The vast majority (Group 1: 67/73, 91.8%; Group 2: 57/59, 96.6%; Group 3: 155/158, 98.1%) also felt friendly and attentively treated. There was no significant difference in the response to the questions between the 3 groups (chi-square test, *P*>.05). Overall, in Group 1, patients were significantly more satisfied with the ophthalmological center when they felt extensively (chi-square test, *P*=.02) and comprehensibly (chi-square test, *P*=.006) counseled. The same was true for patients in Group 1 who agreed completely to the statement that they were friendly and respectfully treated (*P*=.001).

Additionally, patients were asked for areas of possible improvement of the center. Overall, in all groups, roughly 40% (Group 1: 31/76, 40.8%; Group 2: 24/65, 36.9%; Group 3: 72/165, 43.6%) had no complaint (multiple answers were possible). In Groups 2 and 3, 35.4% (23/65) and 31.1% (51/165), respectively, mentioned the center’s organization (multiple answers were possible). In Group 1, the percentage was smaller at 26% (20/76). More patients complained about insufficient communication (multiple answers were possible) in Group 1 (9/76, 11.8%) than in Groups 2 and 3 (3/65, 4.6% and 5/165, 3.0%, respectively; Table 3). More patients mentioned a lack of counseling and information in the center (multiple answers were possible) in Group 2 (7/65, 10.8%) than in Groups 1 and 3 (5/76, 6.6% and 10/165, 6.1%, respectively; Table 3). There were no significant differences between groups.

### Results in Group 1 (App Use)

#### Communication Forms

To ascertain the acceptability of this mode of communication, patients were inquired about their willingness to communicate exclusively through the app. Surprisingly, none of the patients expressed agreement for an exclusively digital communication channel. A significant majority (60/77, 78%) of respondents expressed a preference for a hybrid communication mode, involving both the app and traditional telephone channels. An interesting gender-based trend was observed, with men showing a slightly higher inclination toward this hybrid mode (chi-square test, *P*=.08). A minority (6/77, 8%) of respondents expressed an exclusive preference for the app. Meanwhile, 14% (11/77) of participants expressed a leaning toward solely using the telephone for communication.

#### Software Installation on the Mobile Device

The vast majority (59/73, 81%) of patients in Group 1 handled the process of installation very well. Only 5 (7%) patients saw it as a burden and described the installation process as troublesome. However, half (38/77, 49%) of the patients needed support to install the app. Significantly more male patients (chi-square test, *P*=.03) and people with a higher degree of education at a university (chi-square test, *P*=.004) were able to install the app without external help.

Android was the most prevalent operating system (54/77, 70%). The app was used 50 days on average, and the average use time was 6 minutes 44 seconds per session. The most read articles concerned the type of IOL that can be inserted (312 views) and the postoperative recommendation on patient’s behavior (275 views). Information on the practicing doctors (264 views), the process of surgery preparation (243 views), and the recommendations for this appointment (205 views) were also of interest. The description of technical diagnostic examinations was of less interest (less than 5 views).

#### Satisfaction With the App

**Figure 2:**
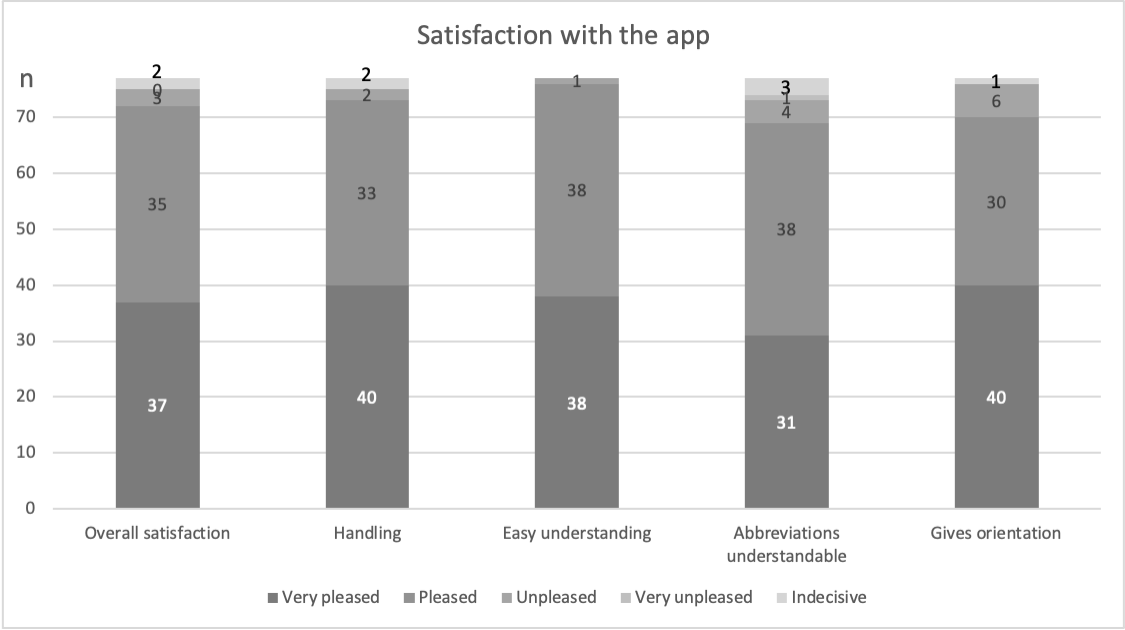
Responses of participants in Group 1 (app use; n=77) regarding app satisfaction after 7 days postoperatively with respect to overall satisfaction, handling, understanding, abbreviations and orientation throughout the perioperative process.

Participants in Group 1 were asked 1 week postoperatively about the overall experience with the app. In general, the users were very satisfied with the experience (very pleased: 37/77, 48%; pleased: 35/77, 46%). Only 3 (4%) patients were unpleased and none of the patients were very unpleased (Figure 2). There was no difference in satisfaction for patients aged under 70 years, those aged between 70 and 80 years, and those aged 80 years or above. The appreciation for the app 1 week postoperatively was greater among patients with a previous vocational education than among patients with a university degree (chi-square test, *P*=.07).

Most patients also agreed that the handling of the app was pleasing (73/77, 95%), and texts and abbreviations were easy to understand (76/77, 99% and 69/77, 90%, respectively). The vast majority (70/77, 91%) also agreed with the statement that the app provides orientation throughout the perioperative process (Figure 2). Patients who received support for the installation were more satisfied with the app within the first 7 postoperative days (chi-square test, *P*=.003).

#### Informational Content of the App

**Figure 3:**
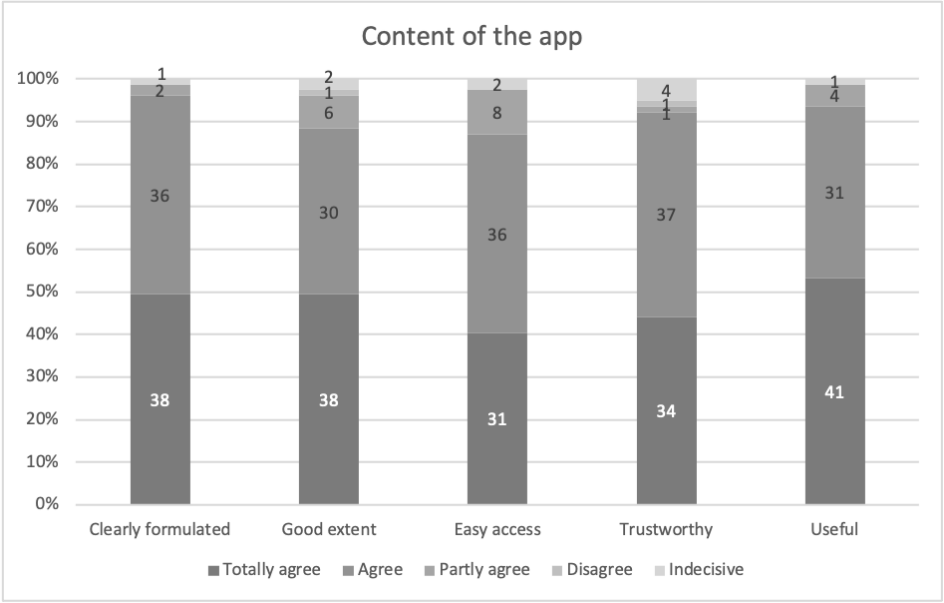
Responses of participants in Group 1 (app use; n=77) regarding app content with respect to clarity, extent, access, trustworthiness, and usefulness.

Patients were also asked about the content provided in the app. Almost all respondents stated that the app is clearly phrased (74/77, 96%), provides sufficient data (68/77, 88%), and provides easy access (67/77, 87%) (Figure 3). Over 90% also agreed that the content is trustworthy (71/77, 92%) and useful (72/77, 94%) during the perioperative process. Patients with a lower degree of education found the content significantly more useful (chi-square test, *P*=.04). Women tended to find the content more useful (chi-square test, *P*=.08). There was no variation in content appreciation across different age groups.

#### Design of the App

**Figure 4:**
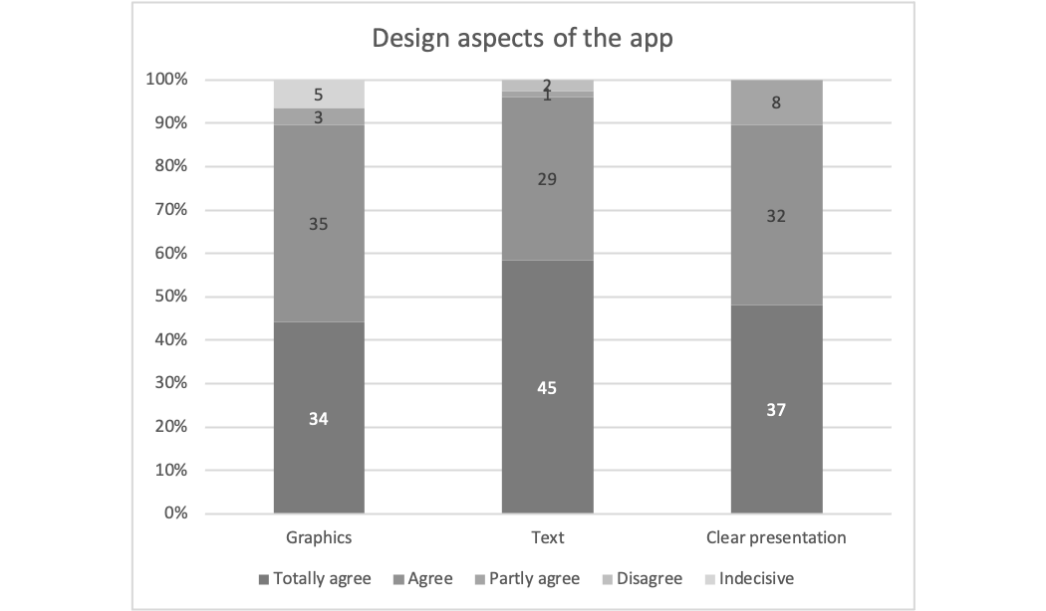
Responses of participants in Group 1 (app use; n=77) regarding graphics, text, and clear presentation of the content in the app.

Patients were also queried regarding the design of the app. Satisfaction with the graphical representations was expressed by 90% (69/77) of patients. A predominant proportion (74/77, 96%) of patients concurred that the textual layout was easily legible. A substantial majority (69/77, 90%) affirmed the clarity in content presentation (Figure 4). No significant variations were observed based on age group or gender.

#### Re-evaluation After 4 to 6 Weeks and Final Suggestions on the Patient’s Journey

Four to six weeks after surgery, patients were asked if the app helped in the whole process. We found that 80% (58/73) of patients stated that the app was helpful. Approximately 20% thought that the app was less helpful (10/73, 14%) or not helpful (1/73, 6%). Patients who had previously used a health app did not appreciate the app significantly more (chi-square test, *P*=.77), but those who had installed the app with help were significantly more satisfied with it (chi-square test, *P*=.02). After 4 to 6 weeks, a higher proportion of female patients expressed appreciation for the app compared to male patients, though the difference was not statistically significant (chi-square test, *P*=.12). Patients with a high degree of satisfaction with the ophthalmological center tended to be more satisfied with the app throughout the cataract surgery journey (chi-square test, *P*=.13). Patients with a lower degree of educational training reported significantly higher satisfaction with the app (chi-square test, *P*=.04), while patients with a university degree reported less satisfaction (chi-square test, *P*=.01).

There were 11 unsatisfied participants, and they were asked why they did not approve of the app. Multiple responses were possible. Five respondents declared that the app did not provide new information. Four mentioned ill functioning of the app or misleading information. Three agreed that the push notifications were disturbing. One patient favored personal contact with a physician.

These respondents were also asked how to improve the app and make it more helpful. Multiple answers were again possible. Four respondents suggested an improvement in the design. Three wanted more personalized functioning of the app, for example, regarding the push notifications. Only 2 proposed an improvement of the informational content and text. Three patients did not provide any suggestions.

Patients who agreed that the app was helpful were also asked how they would improve the app. Eight respondents thought that personalization of the app would be helpful. This mainly concerns the setting of notifications. Four patients desired an improvement of appointment scheduling. Three respondents addressed concerns regarding the font size and contrast of the font and background. Two desired an improvement of the content of the text, and one desired a desktop version of the app.

## Discussion

### Principal Findings

In this study, 297 patients who underwent cataract surgery were enrolled, and 3 different groups were established. An adaptable mobile app was used by 77 patients in Group 1 for at least 2 weeks prior to the pre-examination day and for 4 to 6 weeks after surgery. Group 2 included patients who denied use of the app, and Group 3 included control patients.

In our study, almost all patients who used the app reported good user friendliness and appreciated the digital data provided for the organization, and the scheduling and information of the surgery. Age did not play a major role in the appreciation of the app when agreeing to use the app, although patients in Group 2 were significantly older. Patients who did not have a higher university degree had the most benefits from the informational content of the app and were the most satisfied with the information. Female patients tended to appreciate the information provided more than male patients. However, male individuals and academics are in general more aware of technology and can handle the app more easily. Nevertheless, other individuals can benefit from the use of the app throughout the cataract surgery journey. App users demonstrated a noninferior high satisfaction with the treatment in comparison with patients who were only conventionally informed about the surgery.

### Satisfaction With the Ophthalmological Center

Among all groups, most respondents in this study were very satisfied with the organization and the ophthalmological center where they underwent the surgery. This is in line with the findings of most other studies on the perioperative satisfaction of patients [29-31]. In these studies, outpatients were very content with the information provided, the politeness, the surgical results, and the professional competence.

### Cataract Surgery Satisfaction With a Focus on the Information or Content Provided

In our study, almost all enrolled patients felt well informed during the perioperative process, and there was no significant difference between the 3 study groups. Multiple other studies have confirmed that during the process of a surgical intervention, the degree of information provided is a significant predictive factor of satisfaction [31]. For cataract surgery, preoperative information supplied by a video or other digital content can improve knowledge among patients. The app used in our study included such a video. Pager [32] demonstrated in a randomized controlled trial that preoperative display of a video on the phacoemulsification procedure significantly increases patient understanding of and satisfaction with the cataract surgery while decreasing unease. Patients showed these results independent of past cataract surgery and notwithstanding that, in general, patients stated they had already obtained sufficient information beforehand [32]. Another study showed that information levels were higher in patient groups that received additional information from digital animated videos [33]. However, these videos as well as other information should be thoroughly selected by the ophthalmological center as digital or social media information, such as that on YouTube, might be misleading [34].

### Consideration of Age and Gender in the Use of Digital Devices

In this study, the average age of participating respondents was unsurprisingly high, with a median age of 69 years in Group 1, 79 years in Group 2, and 74 years in Group 3. Age seemed to play no obvious role in Group 1, which was the interventional user group. No evidence was found that age influenced the use of the app or satisfaction with the app. However, patient age in Group 2 (app denial) was significantly higher, indicating that older patients might be hesitant to use digital devices. Male patients tend to be more aware of technology than female patients, and a higher educational background favors easier handling of the app. Another study demonstrated that male elderly people had more ease with the use of the internet and digital devices [35]. This is also true for the use of medical apps. Elderly females seem to be more difficult to reach with these innovative new technologies than males [36]. Perceived serenity and a sense of control while using the app, personal innovativeness, self-perceived effectiveness, and service possibilities were aiding factors to animate mHealth use among elderly communities [36,37].

### mHealth and Higher Educational Background

This study showed that patients with a lower degree of educational training appreciated the content of the app more than those with an academic degree. Patients who do not screen the internet beforehand for consistent information might benefit more, as general understanding and basic knowledge are lower. Studies have evaluated mHealth in diverse ethnic and educational groups, as well as in older and low-income groups. The results showed that mobile devices might be the preferred method of collecting health information from these groups [38]. Patients from low-income backgrounds appreciate the use of mHealth technology to both manage chronic diseases and overall health. As educational and socioeconomic gaps strongly correlate with higher rates of chronic conditions, such as obesity, diabetes, and hypertension, in these communities, mHealth can prove to be an asset [39]. Apps can provide a low threshold source of information and empower people in these communities to improve health outcomes.

### Economic Perspective on mHealth

The willingness to use a mobile phone and the readiness of a mobile phone are prerequisites for establishing mHealth in clinical practice. The use of mHealth should also benefit and facilitate workflows within medical centers. If the physician’s time invested in the patient’s education on the disease and surgical interventions can be reduced while keeping the knowledge and satisfaction levels high, the center can gain in terms of workflow effectiveness. However, integration in the work process of a clinic can be troublesome, and it has been proven to be a burden for both health care professionals and consumers [3,40]. Development of the cataract education app was encouraged by the desire to reduce the number of common questions raised by patients as well as include additional information so that patients’ visits to the ophthalmologist would be optimized without loss of quality and satisfaction. In this study, patients received all relevant information material concerning the app by mail, could download the app, and could inform themselves about the surgery prior to the appointment for surgery pre-examination. Study organizers believe that this might have helped to reduce the time needed for explanation about the app and the surgery for app users. On the other hand, the time needed might have increased for other patients who did not have prior experience with such health care apps before and needed support for the app setup or standard consultation about the surgery. However, patient consultations could have been more efficient once the app was installed and patients could attend postoperative examinations after being informed over the app. Additional human resources were needed for the preparation and adaptation of the informational content of the app and information about the app, as well as for periodic review of the arrangements of patient appointments and entry into the app database. In the future, complete integration of the app within the software of the ophthalmological center should be pursued, so that all relevant data could be synchronized automatically. The employees of the practice also needed educational courses and an adaptation period in order to learn about the content of the app and be able to support patients with app use. Study organizers found this process to be uncomplicated for employees who owned smartphones and had prior experience with other apps and to be time-consuming for others. Patients in this study were reluctant to shift the communication and scheduling of the ophthalmological center to a solely digital mode. However, from the health economics perspective, mHealth has already been demonstrated to be a valuable and effective tool in health care systems and environments with limited resources [41,42]. We believe that short time investments in the development of the app, preparation of information material, and education of employees can provide long-term benefits for the optimization of workflow in the ophthalmological center.

### Limitations

The limitations of this study include the inclusion of patients from a single outpatient health care center, analysis of the app for a single disease treatment, and testing of the app solely in elderly patients. The allocation within this clinical study was not blinded; therefore, the outcome ascertainment might have been influenced by the knowledge of this allocation and furthermore might be evident in the median age of the groups investigated. The study was also limited by the fact that the education of app users was not limited to a solely digital form via the app as they also received conventional information.

### Conclusion

Mobile apps with high user friendliness have the capacity to increase patient knowledge, ensure satisfaction with the treatment, and improve workflows; however, they still face challenges regarding clinical effectiveness, lack of integration in health care delivery, and further validation processes in safety and privacy [9,10]. Further analysis and large multicenter prospective clinical trials are needed to show areas for improvement, prove the potential benefits of apps, and identify specific patient groups that could benefit the most from app use. Future studies could also investigate if apps help to optimize patient management while reducing the duration of consultation, including surveying medical professionals about their experiences with patients using apps. Furthermore, we recommend further developments to synchronize apps with the software of health care centers in order to optimize management and increase the usability of apps.

## Appendix

Multimedia Appendix 1 Demonstration video of the cataract app.

Multimedia Appendix 2 Questionnaires for all groups.

Multimedia Appendix 3 CONSORT-EHEALTH (V 1.6.1) checklist.

## Abbreviations

IOL: intraocular lens
IOP: intraocular pressure
mHealth: mobile health

## Data Availability

All data are available upon request by email to the corresponding author.
