# Supplementary material for: User Friendliness and Perioperative Guidance Benefits of a Cataract Surgery Education App: Randomized Controlled Trial
Source: JMIR Form Res. 2024 Mar 29;8:e55742. doi: 10.2196/55742 (PMC11015376; doi:10.2196/55742)
Supplement: Multimedia Appendix 2 [file formative_v8i1e55742_app2.docx]

**Questionnaire A**

1. **Overall, how satisfied are you with the app?**

Very unsatisfied

Rather unsatisfied

Quite satisfied

Very satisfied

Cannot decide

1. **What do you really like about the app? (Answer to this question is optional)**

Your answer:…………………………………………………………………………………………………………………………………………………

1. **What do you think needs to be improved in the app most? (Answer to this question is optional)**

Your answer:…………………………………………………………………………………………………………………………………………………

1. **Use of the app (to what extent do you agree with the following statements?)**

|  | Not at all | Partly | I agree | Fully | Cannot decide |
| --- | --- | --- | --- | --- | --- |
| 4.1 App is easy to use |  |  |  |  |  |
| 4.2 App uses easy-to-understand terms |  |  |  |  |  |
| 4.3 App uses easy-to-understand abbreviations |  |  |  |  |  |
| 4.4 App gives me good orientation |  |  |  |  |  |
| 4.5 App shows my position in perioperative process |  |  |  |  |  |

1. **Design of the app (to what extent do you agree with the following statements?)**

|  | Not at all | Partly | I agree | Fully | Cannot decide |
| --- | --- | --- | --- | --- | --- |
| 5.1 Graphic explanations are appealing |  |  |  |  |  |
| 5.2 Text design is easily readable |  |  |  |  |  |
| 5.3 Content presentation is clear |  |  |  |  |  |

1. **Content of the app (to what extent do you agree with the following statements?)**

|  | Not at all | Partly | I agree | Fully | Cannot decide |
| --- | --- | --- | --- | --- | --- |
| 6.1 Content is clearly formulated |  |  |  |  |  |
| 6.2 Extent of information is appropriate |  |  |  |  |  |
| 6.3 Important information is easily accessible |  |  |  |  |  |
| 6.4 Information is trustworthy |  |  |  |  |  |
| 6.5 Content is useful |  |  |  |  |  |

1. **Contact (How would you prefer to be contacted?)**

Only via the app  Preferably via the app  Both via the app and telephone

Preferably via telephone  Only via telephone

**Questionnaire B**

1. **Why did you dismiss using the app?**

Your answer:…………………………………………………………………………………………………………………………………………………

**Questionnaire C**

1. **How satisfied are you overall with the practice?**

Very unsatisfied

Fairly unsatisfied

Fairly satisfied

Very satisfied

Cannot decide

1. **The practice (to what extent do you agree with the following statements?)**

|  | Not at all | Partly | I agree | Fully | Cannot decide |
| --- | --- | --- | --- | --- | --- |
| 2.1 I was extensively counseled and informed in the practice |  |  |  |  |  |
| 2.2 I was comprehensibly counseled and informed in the practice |  |  |  |  |  |
| 2.3 I was friendly and attentively treated in the practice |  |  |  |  |  |

1. **What did you particularly like about the treatment in our practice?**

Your answer:…………………………………………………………………………………………………………………………………………………

1. **What did you like less about the treatment in our practice or what should be improved?**

Your answer:…………………………………………………………………………………………………………………………………………………

1. **How helpful was the app during your treatment?**

Not helpful (continue to answer questions 6 and 7)

Less helpful (continue to answer questions 6 and 7)

Rather helpful (continue to answer questions 8 and 9)

Very helpful (continue to answer questions 8 and 9)

Cannot decide

1. **Why was the app not helpful/less helpful for you?**

Your answer:…………………………………………………………………………………………………………………………………………………

1. **What could we improve on the app to make it helpful for you?**

Your answer:…………………………………………………………………………………………………………………………………………………

1. **What did you find particularly helpful in the app?**

Your answer:…………………………………………………………………………………………………………………………………………………

1. **Do you have any suggestions or requests for improvements to the app?**

Your answer:…………………………………………………………………………………………………………………………………………………

**Questionnaire D**

1. **How satisfied are you overall with the practice?**

Very unsatisfied

Fairly unsatisfied

Fairly satisfied

Very satisfied

Cannot decide

1. **The practice (to what extent do you agree with the following statements?)**

|  | Not at all | Partly | I agree | Fully | Cannot decide |
| --- | --- | --- | --- | --- | --- |
| 2.1 I was extensively counseled and informed in the practice |  |  |  |  |  |
| 2.2 I was comprehensibly counseled and informed in the practice |  |  |  |  |  |
| 2.3 I was friendly and attentively treated in the practice |  |  |  |  |  |

1. **What did you particularly like about the treatment in our practice?**

Your answer:…………………………………………………………………………………………………………………………………………………

1. **What did you like less about the treatment in our practice or what should be improved?**

Your answer:…………………………………………………………………………………………………………………………………………………

**Questionnaire E**

1. **Are you a patient yourself or is your partner/friend or relative a patient?**

I am a patient

My partner/friend or family member is a patient

1. **Did you install the app yourself or with the help of other people?**

Myself

With the help of other people

1. **How easy did you find installing the app and signing up? (School grades 1-6, where 1- is very easy and 6- is very complicated)**

1- Very easy

2- Easy

3- Satisfactory

4- Sufficient

5- Complicated

6- Very complicated

1. **Have you already used another healthcare app before (for example of your health insurance company)?**

Yes

No

1. **What are your hobbies?**

Your answer:…………………………………………………………………………………………………………………………………………………

1. **What is your highest level of education?**

No degree

Elementary or secondary school

Secondary modern school

High school

Vocational training

Technical college

University (bachelor degree)

University (master´s degree)

University (doctorate)

**Questionnaire F**

1. **Have you already used another healthcare app before (for example of your health insurance company)?**

Yes

No

1. **What are your hobbies?**

Your answer:…………………………………………………………………………………………………………………………………………………

1. **What is your highest level of education?**

No degree

Elementary or secondary school

Secondary modern school

High school

Vocational training

Technical college

University (bachelor degree)

University (master´s degree)

University (doctorate)
